# Adolescents’ Perceptions of Their Problematic Use of ICT: Relationship with Study Time and Academic Performance

**DOI:** 10.3390/ijerph18126673

**Published:** 2021-06-21

**Authors:** Adoración Díaz-López, Ana Belén Mirete-Ruiz, Javier Maquilón-Sánchez

**Affiliations:** 1 Didactics Department and School Organization, Faculty of Education, University of Murcia, 30100 Murcia, Spain; adoracion.diaz@um.es; 2 Methods of Educational Research and Diagnosis in Education, Faculty of Education, University of Murcia, 30100 Murcia, Spain; anabelen.mirete@um.es

**Keywords:** problematic use of ICT, adolescence, mobile, videogame, academic performance, family supervision

## Abstract

Today, the use of Information and Communication Technologies (ICT) is part of the daily lives of adolescents. However, its widespread use in all areas, the vulnerable condition of adolescents and the imminent consequences of problematic use are awakening a growing social and educational concern. With the purpose of looking into this problem, the following research aims are formulated: (1) Analyse the perception of adolescents about their academic performance and the interference of ICT in their development; (2) Describe the frequency of use of ICT and its influence on study time and grades; and (3) Analyse the relationship between family supervision of ICT and academic performance. The representative sample consisted of 1101 adolescents from 10 educational centers in the Southeast of Spain. Descriptive statistics, contingency tables, Chi Square, Cramer’s V and Linear Regression were calculated. The results show that more than 50% of the students believe that they would spend more time studying if they did not have continuous access to technologies. Likewise, 20% of the students identify ICT as responsible for the decline in their academic performance. Statistically significant relationships were found between time limitations for Internet access and academic performance. It is therefore concluded that the problematic use of ICT in adolescence is a phenomenon that demands intervention, and the training of parents and adolescents in the responsible use of ICT is urged.

## 1. Introduction

In recent years, ICTs have become an essential tool within the educational field, the virtual teaching modality called “digital classrooms”, or the school 2.0 program [1,2], where tablets or laptops are used as the main element in the teaching-learning process, and as a means of accessing knowledge in the school context. In fact, according to [3] at present, ICTs are the fundamental resource in the teaching and learning processes and in the leisure of minors, which with the appearance of COVID-19, has become even more accentuated.

There are many and very diverse studies that analyse the contribution of ICT to the improvement and innovation of training processes in the classroom [3,4,5,6,7], ignoring the negative consequences that its abuse can cause at an academic level. In recent years, however, the scientific community has been alerted by this problem [8], verifying that the problematic use of ICTs maliciously extends to all contexts of the lives of young people [9], with a growing influence in the school environment aggravated by the presence of mobile telephony at increasingly younger ages, since according to the National Institute of Statistics (INE) [10], 25% of 10-year-old school children have access to a mobile device, 45% at 11 and more than 90% at the age of 14 and older. At an international level, the last systematic review of this topic showed that the prevalence of ICT use among adolescents is surprisingly high; over 90% of teenagers in the USA and Japan and 72% in China use Internet on a daily basis, while the Internet’s extensive prevalence has exceeded 20% for Indian and Iran teenagers [11]. 

This approach conceptualises the problematic use as the inability to control the use of cyberspace, generating discomfort from abstinence and negative repercussions in everyday life [12]. Other authors defined it as actions related to the abandonment of commitments of a family, educational and social nature, due to the need to be connected to ICT, a decrease in academic performance, putting virtual relationships before real social contact, states of stress, restlessness or anxiety due to being deprived of technology or the inability to communicate, or rest disturbances and unjustified disruptive behaviors such as aggressiveness or irritability when interrupted while using technology (mobile phone, console, others) [13]. According to gender, girls show more problems dealing with problematic use of mobile phones, while boys present a higher problematic use of console [13].

It has been shown that ICTs themselves are resources with unlimited potential, and they can be extremely attractive and valuable at the school level. In this sense, coinciding with [14], we can affirm that the responsible use of ICT positively correlates with the improvement of academic performance in certain subjects. Nevertheless, not all the agents involved in the process are prepared to exploit, rationally and in a beneficial way, the potential of technological resources. Adolescence is an especially vulnerable stage, in which ICT is used without prior training [15], and almost without family supervision [16,17], which can have undesirable consequences in the school dynamics of puberty [8], as it is proven that a lack of parental control strengthens the tendency to present problematic use of ICT [18].

In this sense, [19] points to ICT and mobile phones as the main distractors of this stage, which in addition to being used by adolescents at all hours (30%) and a lot (34%) [13], promote the displacement of young people’s attention away from school responsibilities [20]. Along the same lines, according to [21], 28% of students point to ICT as the main distracting element in their teaching and learning process. 

This inattention is aggravated by the constant technological overexposure to which adolescents are subject to daily. Following this idea, [6] warns that the strong cognitive stimulation resulting from long periods of exposure to screens is counterproductive when it comes to being employed in academic responsibilities. In this regard, recent studies [22] warn of the detriment of both academic and cognitive performance of school-age children due to the problematic use of ICT. With a similar approach, [23] bases this fact on the fact that several hours a day in front of the screens cause problems among the younger population, when it comes to staying focused on tasks that require dedication and sustained attention over time, from a proactive attitude.

Another worrying aspect is related to the decrease in grades. In this line, [24] claims that the incessant growth in the use of ICT has led students to neglect their academic responsibilities, which negatively influences their grades. Correlation between the frequency of use of ICT and grades have been found by [6], underlining that students with the worst academic results showed a higher consumption of mobile telephony and videogames. Along the same lines, other authors [25,26] blame video game consoles as the main cause of demotivation towards academic tasks, and warn that a high gaming frequency is inversely related to academic performance.

Such is the presence of ICT in school life, that as [27] suggests, 14% of students acknowledge having decreased their involvement and dedication to studies due to the need to invest more time in front of screens. In this regard, [8] points out that 44% of teenagers recognise that the use of ICT makes them lose the time that they could dedicate to academic purposes.

From this general, national and international framework, which justifies the importance of the subject under study, and with the delimitation that has been made of the problem, after reviewing the literature, it is found that the maladaptive use of ICT is one of the main social, educational and family problems among young people [11]. Above all, however, it revealed that the consequences are already visible in society from its broader perspective.

For this reason, we have considered presenting this research that looks into how the problematic use of ICT is affecting the academic performance of adolescents. In our study, we will stablish as criteria for the problematic use of ICT the following: on one hand, the perception of students about ICT as distractors of academic duties; ICT as tools that negatively affect academic performance; frequency of mobile phone use, console and other devices with Internet connection; abandonment of academic tasks due to being online for longer; need to invest more and more time on ICT; staying up late using ICT; and study time spent on the Internet.

For this purpose, the following specific aims are formulated: (1) Analyse the adolescents’ perception of their academic performance and the interference of ICT in their development; (2) Analyse the factors that predict the decline in academic performance; (3) Describe the frequency of use of ICT and its influence on dedication to schoolwork and academic performance; and (4) Analyse the relationship between family supervision and academic performance and grades. The following hypotheses are formulated:

**H1.**  *Frequency of use of mobile phone, abandonment of academic tasks due to being online for longer, need to invest more and more time online, staying up late using mobile, study time spent on the Internet, age and stress predict the decline in academic performance due to the mobile phone use.*

**H2.**  *Frequency of use of console, abandonment of academic tasks due to being online for longer, need to invest more and more time online, staying up late using console, study time spent on the Internet, anger when playing is interrupted, age and stress predict the decline in academic performance due to game console use.*

**H3.**  *Frequency of use of ICT interference in study time and grades.*

**H4.**  *Family supervision of access to social networks and time limitations of Internet access are related to a better perception of academic performance.*

## 2. Materials and Methods

### 2.1. Participants

The selected sample was made up of 1101 participants, of whom 47.8% were boys and 52.2% girls, enrolled in Grade 7 (32.4%), Grade 8 (22.3%), Grade 9 (24.3%) and Grade 10 (21.0%), and aged between 11 and 18 years (17% between 11 and 12; 46.1% between 13 and 14; and 36.2% between 15 and 18). The sample was collected from an intentional non-probabilistic sampling process from ten publicly owned educational centers in Murcia (Spain). For the representative selection of the sample, a territorial division of the nine counties that make it up was established, and centers belonging to each were randomly selected. It was not necessary to establish exclusion criteria for the sample due to the nature of the study.

### 2.2. Design

We use a quantitative non-experimental survey-type design. A transactional design was used; therefore, the study establishes its focus of inquiry at a specific moment [28].

### 2.3. Instruments

The Ud-TIC questionnaire was used (Maladaptive Use of ICT) (Appendix A), designed and validated ad hoc from instruments previously validated for the Spanish-speaking adolescent population. Specifically, these are the CERI (Questionnaire of experiences related to the use of the Internet), CERM (Questionnaire of experiences related to the use of mobile phone) [29] and CERV scale (Questionnaire of experiences related to the use of videogame) [30]:

The instrument comprises two dimensions:

(1) Dimension 1. Sociodemographic data:

This consists of seventeen items. It contains questions about personal data (age, sex, course and region), academic performance: grades in instrumental areas (the marks they got in the last trimester), coded as insufficient (1–4), good (5–6), remarkable (7–8) or outstanding (9–10). Three questions about attitude towards the study and interference of ICT in it (Likert scale from 1 to 5), family supervision (dichotomous response), adult in charge of supervision (closed polytomous response), hours or availability of access to ICT (closed polytomous response) and stress in the absence of ICT (dichotomous response). 

(2) Dimension 2. Problematic use of ICT:

This consists of nineteen items, three of which are related to the frequency of use of video games (1. Never; 2. Hardly ever; 3. Only during the weekend; 4. Several times a week; and 5. Every day), mobile phone (1. Never; 2. Only when I need it; 3. Frequently; 4. A lot; and 5. At all hours) and other devices with an Internet connection (1. Never; 2. Only when I need it; 3. Frequently; 4. A lot; and 5. At all hours) and the remaining sixteen address experiences related to the problematic use of mobile phones, the Internet and video games (this is a Likert scale with response options from 1 to 5: 1. Never; 2. Sometimes; 3. Almost always; 4. Quite a few times; and 5. Always).

The reliability of the instrument Ud-TIC is very high (α = 0.841). Regarding the construct validity, a 6-factor model is reached that explains 64.27% of the variance: Factor 1, Experiences related to the problematic use of the mobile pone; Factor 2, Experiences related to the problematic use of the console; Factor 3, Abandonment of tasks and avoidance of problems; Factor 4, States of irritability; Factor 5, Console use frequency; and Factor 6, Sleep disturbances (KMO = 0.813; Sig < 0.005). It concludes by pointing to the Ud-TIC as a valid and reliable instrument for being used with a Spanish-speaking adolescent population. 

### 2.4. Procedure

The procedure follows the generic phases and tasks of any research process. The following aspects stand out due to their relevance: in accordance with rule 8.2 of the Parents’ Association (APA), authorisation was requested from both parents of the participants and an informed consent document was delivered. In the same way, informed consent documents were delivered to each of the students, which included information on the anonymous and voluntary nature of their participation. Both documents had the endorsement of the Research Ethics Commission. The information collection instrument was applied in paper format and in person. The information collection was carried out during the tutoring hours, and the group’s tutor collaborated. The duration of the completion of the instrument ranged between 12 and 20 min.

### 2.5. Analysis of Data

A descriptive analysis of the sample was carried out, in which the central tendency indices (mean and standard deviation) were calculated. Likewise, the Kolmogorov-Smirnov (K-S) normality test was performed, determining that the sample does not follow a normal or symmetric distribution (*p* ≤ 0.05), but confirming that one of the limitations of this test (KS) is its conservative tendency, making the non-normality hypothesis accepted in all situations (for n = 1000 subjects). Moreover, the Lilliefors correction (KSL) was applied, which does allow a certain percentage (3.7% for samples of n = 1000 subjects) rejecting the null hypothesis, but in this case the existence of an asymmetric distribution was reiterated. Therefore, non-parametric statistics such as the Mann-Whitney U (two groups) and Kruskal Wallis (more than two groups) were applied.

In the aim that analyses the relationship between variables, contingency tables and Pearson’s Chi square were used to determine the existence of statistically significant relationships. Cramer’s V test was used to assess the magnitude of the associations between the variables. Finally, a linear regression analysis was performed to analyse the predictive value of the study variables. 

## 3. Results

### 3.1. Adolescents’ Perception of Their Academic Performance and the Interference of ICT in Their Development

In the first of the aims, which analysed the perception of adolescents about their academic performance and the interference of ICT in their development, it was found that 47% of respondents fully agree with the statement that they could get better grades, followed by 36% who strongly agree (M = 4; Sd = 0.906). Regarding the time they dedicate to study, 30% of the students maintain that they do not invest enough time, compared to another 30% who say that they spend enough time and 40% who declare themselves indifferent (M = 3; Sd = 1.056). 

In addition, 35% maintain that the use of the Internet, “always”, “almost always” or “quite a few times” negatively affects their academic performance (M = 4; Sd = 1.219), and that the use of the mobile device has a negative impact on the performance of 28% of respondents (M = 4; SD = 1.166). In this way, 22% recognise that for “always”, “almost always” or “quite a few times”, they abandon their academic tasks to spend more time connected to the Internet (M = 4; Sd = 0.934) or playing video games (Md = 5; Sd = 1.302).

Regarding the impact of the Internet and mobile device use on academic performance, no statistically significant differences were found according to sex (*p* = 0.602), nor were these differences detected in the abandonment of academic tasks for preferring to be connected to the Internet (*p* = 0.078). However, regarding the abandonment of school obligations to play video games, boys are the notably more vulnerable group, with an incidence in 28% of cases, compared to 19% of girls. In this case, statistically significant differences were found between the abandonment of tasks due to the need to play the video game console and sex (*p* ≤ 0.001).

When the course variable was considered, it was obtained that, in terms of the interference of ICT in academic performance, it should be noted that statistically significant differences were found according to the course – being, in both cases, Grade 10 students who suffer a greater negative impact on their performance due to the use of the Internet and the mobile (*p* ≤ 0.005); (*p* ≤ 0.001), respectively.

Related to the abandonment of academic tasks to be connected to the Internet, no statistically significant differences were found according to the course (*p* = 0.078). When it comes to playing video games, however, the Grade 9 students are the group with a higher percentage of homework abandonment. Thus, 34% of them acknowledge that they abandon their academic tasks to spend more time playing video games, followed by 22% of Grade 7 and Grade 10 students, and 17% of Grade 8 students (*p* ≤ 0.001).

### 3.2. Analysis of the Factors That Predict the Decline in Academic Performance

Likewise, to determine which factors predict the negative impact of mobile phone use on academic performance, a multiple linear regression analysis was carried out (Table 1). The model carried out explains 30% of the variance, and predicts which factors influence the perception of the negative impact of the mobile phone on academic performance: time spent studying (*p* ≤ 0.001); frequency of mobile phone use (*p* = 0.003); abandonment of tasks due to being online for longer (*p* ≤ 0.001); need to invest more and more time on the mobile phone (*p* ≤ 0.001); staying up late using mobile (*p* = 0.015); and study time spent on the Internet (*p* ≤ 0.001). Nevertheless, neither age nor stress predict the decline in academic performance due to mobile phone use.

As for the console, to determine which factors predict its negative impact on academic performance, a multiple linear regression analysis was carried out (Table 2). The model carried out explains 17% of the variance and predicts which factors influence the perception of the negative impact of the console on academic performance: time spent studying (*p* = 0.013); frequency of console (*p* = 0.009); abandonment of tasks due to being playing for longer (*p* = 0.010); need to invest more and more time on the console (*p* ≤ 0.001); anger when playing is interrupted (*p* ≤ 0.001); and study time spent on the Internet (*p* ≤ 0.001). Nevertheless, age, stress and staying up late to play do not affect or predict the perception of the negative impact of the console on adolescents’ academic performance.

### 3.3. Frequency of Use of ICT and Interference in Study Time and Grades

In the second of the aims, the frequency of ICT use and interference in study time and grades was analysed. When assessing the relationship between frequency of use of ICT and the academic qualifications in the instrumental areas of English, Spanish language, mathematics and social sciences, it was based on the analysis of the interference of the mobile device. Thus, 28.2% of the students who present a high frequency of mobile use (“a lot” or “at all hours”) failed the subject of English, and only 10.8% achieved an outstanding grade; while when the frequency of use of the Smartphone is adapted (“frequently”), the percentage of failures is reduced to 18.5% and 15.6% of this group obtained an outstanding score, and the statistically significant relationship is appreciated among the frequency of use of mobile phone and the grades in English, although the magnitude of the association is low ($x^{2\;}$(12) = 32.09, *p* ≤ 0.05; V = 0.000). Very similar results were obtained in mathematics, where 30.2% of those students who use the mobile in a maladaptive way failed, and only 11.8% achieved the score of outstanding, while by reducing the frequency of use from mobile to normalised use, the percentage of failures is reduced to 17.8%, and that of outstanding ones rises to 16.7%. The data point to a statistically significant relationship, while the magnitude of the association is low ($x^{2\;}$(12) = 35.46, *p* ≤ 0.05; V = 0.000). A statistically significant relationship was also found between the frequency of mobile use and the grades in social sciences or social sciences, as the magnitude of the association between both was low ($x^{2\;}$(12) = 31.72, *p* ≤ 0.05); V = 0.000), so the 30.8% of the group of students who use a mobile phone with a maladjusted frequency (“a lot” or “ at all hours”) present insufficient grades, and only 13.8% achieved an outstanding grade. On the contrary, when Smartphone consumption is adapted (“frequently”), we find that the percentage of students who fail is reduced to 16.8%, and 17.5% reach grades of outstanding. Finally, the high frequency of mobile device use also has negative effects on language subjects. In this sense, 26.2% of minors who make extensive use of the mobile device and with an inappropriate frequency have an insufficient grade, and 9.5% obtained an outstanding grade, while in the group of students who use it with a normal frequency, the percentage of failure corresponds to 23%, and that of outstanding amounts to 15.6%. The data point to a statistically significant relationship between the frequency of ICT use and the grades in the language subject, but the Cramers’ V coefficient determined that the magnitude of the association is low ($x^{2\;}$(12) = 27.10, *p* ≤ 0.05; V = 0.000).

If the technological tool to assess is a game console, it should be pointed out that a statistically significant relationship was found between the frequency of maladaptive use of the console and low grades in the area of mathematics ($x^{2\;}$(12) = 23.296, *p* ≤ 0.05; V = 0.000). Thus, 33.3% of adolescents who play video games daily failed math, compared to 19% of those who play exclusively during the weekend. Additionally, it is necessary to underline that, even though statistically significant relationship between frequency of use of console and grades were not found in any cases, apart from math, in all the subjects analysed, those students who use the game console every day have worse academic results than those who only use it at weekends. Thus, in the subject of English, 31.9% of students failed in the group of those who play daily and 20.3% in the weekend players ($x^{2\;}$(12) = 14.528, *p* = 0.268; V = 0.268); in language, 27.5% failed in regular players and 22.4% in weekend players ($x^{2\;}$(12) = 20.146, *p* = 0.064; V = 0.064); and finally, in the matter of social sciences, 31.2% of failures were established among those who play daily, and only 18.8% among those who play during the weekend ($x^{2\;}$(12) = 18.913, *p* = 0.091; V = 0.091).

**Figure 1:**
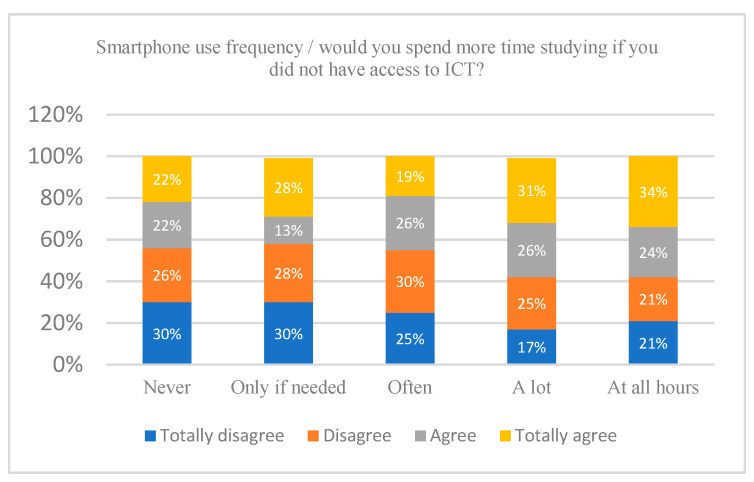
Interference of the frequency of use of the Smartphone in the time dedicated to studying. Note: The figure shows how smartphone use frequency is related to the time teenagers dedicate to study. Axis Y: Would you spend more time studying if you did not have access to the Internet? Axis X = Smartphone use frequency.

When assessing the relationship between frequency of use of ICT and study time, it was found that 60% of the subjects who use the mobile phone “a lot” and “at all hours” fully agree that they would devote more time to studying if they did not have access to ICT. However, as shown in Figure 1, the percentage of ICT interference in the study time is reduced when the frequency of mobile use is lower. The data point to a statistically significant relationship between the frequency of mobile use and the interference of ICTs in the study time, the Cramers V´coefficient determined that the magnitude of the association between both is low ($x^{2}$(16) = 36.444, *p* ≤ 0.05, V = 0.005).

Along these lines, 30% of students who use a game console everyday fully agree with the statement that they would spend more time taking care of academic aspects if they did not have access to ICT, in general, and to a game console, in particular. Likewise, this opinion is shared by 25% of the students who use a game console several times a week. However, when the consumption of video games is restricted to weekends, only 20% of the young people recognise the interference of ICT in their study time. In this way, a statistically significant relationship is appreciated, while the magnitude of the association is low ($x^{2\;}$(16) = 41.588, *p* ≤ 0.001, V = 0.000). 

### 3.4. Family Supervision and Its Relationship with Academic Performance and Grades

In the last of the aims, the relationship between family supervision and academic performance and grades was analysed. For this purpose, two types of family supervision were established: 1. Supervision when connecting to the Internet and using social networks; 2. Time limitations of Internet access. It was found that 56.3% of the young people surveyed connect to the Internet and make use of social networks without supervision by responsible adults. Likewise, it is confirmed that 60.9% of the minors surveyed have unlimited access to the Internet throughout the day.

Along these lines, 40% of students who do not have family supervision when they access the Internet and social networks admit that their academic performance has been negatively affected by Internet use, always (4.5%), almost always (7%), many times (8.2%) or several times (19.8%); while in the group of students with family supervision, only 28% confessed to having interferences in their academic performance due to the use of ICT. The data confirm a statistically significant relationship between the lack of family supervision regarding access to the Internet and social networks and the perception of low academic performance, as well as that the magnitude of the association is moderate ($x^{2\;}$(8) = 15.185, *p* ≤ 0.05, V = 0.551).

**Figure 2:**
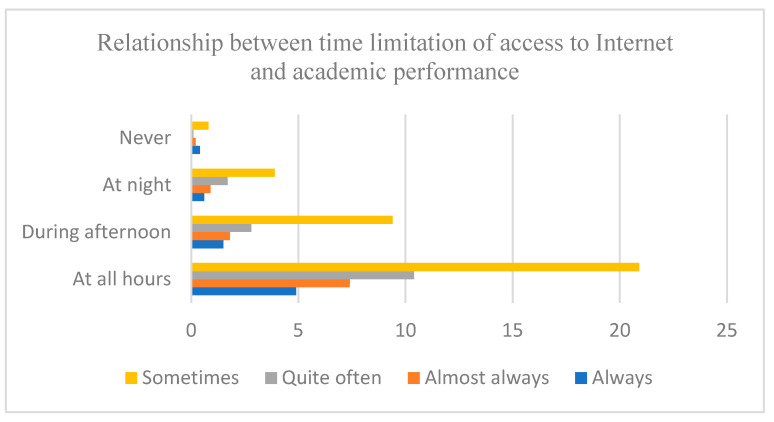
Relationship between time limitation of access to Internet and academic performance. Note: Y axis: When do you have Internet access? Axis X = Academic performance negatively affected by the use of Internet.

When considering the importance of time limitations on Internet access, a statistically significant relationship was found between the limitation of Internet access and the perception of the negative impact in academic performance. The magnitude of the association was low ($x^{2\;}$(12) = 29.308, *p* ≤ 0.05, V = 0.004), and it is highlighted that adolescents who have access to the Internet throughout the day are those with the worst perception of the negative impact in academic performance (Figure 2). Thus, 23% of students who have unlimited access to the Internet indicate that their relationship with Internet always (4.9%), almost always (7.4%) and quite often (10.4%) has a negative effect on their academic performance. However, only 6% of students who have access in the afternoon and 3% of those who have access at night relate the use of Internet with the decrease in school performance. 

As for the grades, it was found that in all cases, students who do not have family supervision when connecting to the Internet and using social networks present worse academic grades in the four subjects analyzed. Thus, the 26.7% of the students who do not have family supervision failed the subject of language, while only the 19% of the students who have family control failed language, meaning a statistically significant relationship was found between limitation of Internet access and academic grades in language, and the magnitude of the association was low ($x^{2\;}$(3) = 10.531, *p* ≤ 0.05, V = 0.015). Very similar results were obtained in the social sciences subject; where 29.2% of those students who do not have family supervision failed, by having familiar control of the use of ICT, the percentage of failures is reduced to 17.9%. A statistically significant relationship was found between limitation of Internet access and academic grades in social sciences, and the magnitude of the association was low ($x^{2\;}$(3) = 21.707, *p* ≤ 0.001, V = 0.000). A statistically significant relationship was also found between the family supervision and the grades in English, as for the magnitude of the association between both was low ($x^{2\;}$(3) = 16.750, *p* ≤ 0.001; V = 0.001), so the 25.8% of the group of students who have family supervision present insufficient grades, while when there is family supervision, only 19.2% of the students fail English. Finally, the family supervision also has positive effects on the mathematic subject. In this sense, 27.2% of the minors whose parents do not supervise the use of Internet present insufficient grades, while the percentage is reduced to 20.8% among those teenagers whose parents supervise their consumption of Internet. No statistically significant relationship was found between family supervision and grades in math ($x^{2\;}$(3) = 6.079, *p* = 0.108; V = 0.108).

## 4. Discussion

The emergence of ICTs in all contexts of young people’s lives has led to a growing dedication of time and attention to their use, and so this time and attention has been taken from other tasks, such as studying. In this situation, the impact of the widespread use of ICT exceeds the limits of academic qualifications, and this extends to the attitude, dedication, and commitment that young people present towards the teaching-learning process.

Regarding school performance, it has been found that the use of the Internet negatively affects academic performance in two out of every ten students, coinciding with what was found by [6], who point out that the widespread use of ICT causes the decline and detriment of performance. In addition, more than 20% admit to abandoning academic tasks to spend more time online. These results differ from those obtained from the study by [27], which place the percentage of incidence at 12.5%, but are coincident with the results of [21], who concluded that the 28% of the students point to ICT as a distractful element in their teaching and learning process. 

In this sense, it was found that the following factors predict the negative impact of mobile phones on academic performance: frequency of use of mobile phone, coinciding with [6], who point out that the high frequency of use of mobile phone causes a negative impact in the academic performance of teenagers; abandonment of academic tasks due to being online for longer than needed; need to invest more and more time online; staying up late using mobile and study time spent on the Internet, coinciding with what was found by [20], who determined that most students who spend their study time online present a worse academic performance. This leads us to reject hypothesis one (H1), since it included age and stress as predictors of academic decline.

As for the console, it was found that the following factors predict the negative impact of console on academic performance: frequency of use of console coinciding with [26], who determined that high frequency of videogames is related to poor academic performance; abandonment of academic tasks due to being playing for longer; need to invest more and more time online; staying up late using console; study time spent on the Internet coinciding with [25,31], who highlight that students who spend more time playing videogames present worse academic performance; anger when play is interrupted; age; and stress. This result implies the rejection of H2, since it includes age, stress and staying up late as predictors of academic decline.

Regarding the frequency of use, it has been confirmed that two-thirds of adolescents who use a mobile phone a lot or always confess that they would spend more time studying if they did not have access to ICT. In this sense, it has been found that when the frequency of the mobile phone use is reduced, the study time increases, coinciding with [6], who found that the students with the worst academic results showed a higher consumption of mobile telephony. Along these lines, the problem extends to video game consoles, since one-in-three students spend their time intended for studying on games and entertainment. These results are consistent with those of [6], who determined that the extended use of the game console displaces the time for studying. Regarding sex, boys present greater interference of the game console in their development as students. These results are like those obtained by [30], who conclude that interference of the game console in academic performance occurs more in boys than in girls. When identifying the influence of the academic course on the problem, it is pointed out that the population group of adolescents in Grade 9 is shown to be the most affected, since one-in-three confess to abandoning or not starting school tasks to play video games.

Regarding the impact of the frequency of use of ICT on grades, the results presented confirm what was indicated by [26,31], who found a statistically significant relationship between the frequency of use of the video console and low academic grades. Therefore, it is concluded that a greater number of hours of play is positively and significantly related to a low academic performance. Regarding the frequency of mobile use, coinciding with [32,33], it was confirmed that those students who make wider use of mobile devices present worse results in all the subjects analyzed, thus confirming the existence of an inverse correlation between the frequency of mobile use and academic results. The higher the frequency of use, the worse the academic results. Therefore, hypothesis three (H3) is accepted.

Likewise, it is concluded that more than half of adolescents use the Internet and social networks without supervision by responsible adults, and even more worrying, almost two out of five do not have time limitations when connected to the Internet. This trend confirms the results published in other studies such as [13,17]

It is proven that limitation of Internet access affects the perception of the negative impact of ICT in academic performance, since teenagers that have access to the Internet during the whole day have a worse perception of the influence of ICT in their academic development than the ones who only have access during the afternoon or only at night. Therefore, hypothesis 4 (H4) is accepted. Finally, it should be emphasized that students who do not have familiar supervision present worse academic grades in all areas than the ones they have familiar control of the use of ICT.

## 5. Conclusions

It is concluded that a third of adolescents feels that the use of ICT has a negative impact on their academic performance. The mobile phone is identified as the most problematic device for young people when it comes to dedicating themselves to their academic tasks. This issue gets even more worrying if the current worldwide situation is considered. Due to COVID-19, and the fact that most of the children have no other choice for learning and socializing, more of their time is spent using ICT [34].

From here, it is considered that it is convenient to also warn of the seriousness of the results obtained from the study regarding the interference of the frequency of use of ICT in the qualifications, since in all cases the high frequency of use is related to the decline in grades in the instrumental areas. In addition, it has been proven that family supervision, both Internet access and social networks, as well as restrictions on Internet access hours are related to academic performance.

It is necessary to alert parents, teachers and society in general that the dynamics of problematic use of ICT in adolescence can have serious consequences on the academic performance of young people, and lead to lower grades. It is therefore urgent to reflect on the state of the issue and design preventive and intervention measures to curb the problematic use of ICTs in adolescence, since although ICTs are a reality that has come to stay, it is also a tool about which we must be trained in its correct or proper use.

## 6. Study Limitation

As for the limitation of the study, it would be appropriate to get deep and more rigorous data in order to ask for the number of hours adolescents use ICT, instead of their perception. Furthermore, academic performance could be measured through actual student ratings, and not through self-reported measures, which may be biased by social desirability. Finally, a study case would be recommended so some other variable can be considered in this dynamic. Regarding the future lines of this study, it is urged to create a program to promote the adapted use of ICT in adolescence, allowing young people to explore the full potential of ICT, away from problematic use.

## Figures and Tables

**Table 1:** Factors that predict the decline in academic performance due to mobile phone use.

	R²	F	B	SE B	β	T	*p*
Model 1	0.291	55.941					0.000 ***
(Constant) Perception of the negative impact of mobile phone on academic performance			2.767	0.286		9.667	0.000
(Predictor) Age			−0.049	0.043	−0.030	−1.138	0.255
(Predictor) Time spent studying			0.212	0.029	0.192	7.203	0.003
(Predictor) Frequency of mobile phone use			−0.090	0.030	−0.083	−2.977	0.003
(Predictor) Abandonment of tasks due to being online for longer			0.221	0.038	0.169	5.545	0.000
(Predictor) Need to invest more and more time on the mobile phone			0.131	0.038	0.105	3.454	0.000
(Predictor) Stress			0.110	0.070	0.040	1.422	0.155
(Predictor) Staying up late using mobile			0.069	0.028	0.071	2.443	0.015
(Predictor) Study time spent on the Internet			−0.264	0.024	−0.289	−11.070	0.000
Model 2	0.289	73.988					0.000 ***
Constant) Perception of the negative impact of mobile phone on academic performance			2.768	0.257		10.774	0.000 ***
(Predictor) Time spent studying			0.216	0.029	0.195	7.364	0.000 ***
(Predictor) Frequency of mobile pone use			−0.099	0.030	−0.091	−2.312	0.001 **
(Predictor) Abandonment of tasks due to being online for longer			0.221	0.038	0.177	5.895	0.000 ***
(Predictor) Need to invest more and more time on the mobile phone			0.137	0.037	0.110	3.664	0.000 ***
(Predictor) Staying up late using mobile			0.069	0.028	0.071	2.443	0.015 *
(Predictor) Study time spent on the Internet			−0.265	0.024	−0.291	−11.159	0.000

Note: significance value: *** = 0.000; ** = 0.001; * > 0.001.

**Table 2:** Factors that predict the decline in academic performance due to console use.

	R²	F	B	SE B	β	T	*p*
Model 1	0.171	25.090					0.000 ***
(Constant) Perception of the negative impact of the console on academic performance			2.227	0.328		6.798	0.000 ***
(Predictor) Age			0.073	0.052	0.040	1.404	0.161
(Predictor) Time spent studying			0.084	0.035	0.069	2.395	0.017 *
(Predictor) Frequency of console use			−0.082	0.031	−0.081	−2.623	0.009 **
(Predictor) Abandonment of tasks due to being online for longer			0.075	0.030	0.075	2.530	0.012 *
(Predictor) Anger when play is interrupted			0.159	0.039	0.141	4.126	0.000 ***
(Predictor) Need to invest more and more time on the console			0.232	0.045	0.172	5.104	0.000 ***
(Predictor) Stress			−0.003	0.081	−0.001	−0.040	0.968
(Predictor) Staying up late playing			0.044	0.033	0.041	1.328	0.185
(Predictor) Study time spent on the Internet			−0.154	0.029	−0.153	−5.407	0.000 ***
Model 2	0.169	37.077					0.000 ***
(Constant) Perception of the negative impact of the console on academic performance			2.488	0.278		8.955	0.000 ***
(Predictor) Time spent studying			0.085	0.034	0.070	2.485	0.013 *
(Predictor) Frequency of console use			−0.086	0.031	−0.085	−2.804	0.005 **
(Predictor) Abandonment of tasks due to being online for longer			0.076	0.030	0.077	2.572	0.010 **
(Predictor) Anger when play is interrupted			0.168	0.038	0.149	4.418	0.000 ***
(Predictor) Need to invest more and more time on the console			0.241	0.045	0.179	5.419	0.000 ***
(Predictor) Study time spent on the Internet			−0.159	0.028	−0.157	−5.620	0.000 ***

Note: significance value: *** = 0.000; ** = 0.001; * > 0.001.

## Data Availability

The data that support the findings of this study are available from the corresponding author, upon reasonable request.

## Appendix A

The Ud-TIC questionnaire
